# Peptides Derived From S and N Proteins of Severe Acute Respiratory Syndrome Coronavirus 2 Induce T Cell Responses: A Proof of Concept for T Cell Vaccines

**DOI:** 10.3389/fmicb.2021.732450

**Published:** 2021-09-24

**Authors:** Yu-Sun Lee, So-Hee Hong, Hyo-Jung Park, Ho-Young Lee, Ji-Yeon Hwang, Seo Yeon Kim, Jun Won Park, Kang-Seuk Choi, Je Kyung Seong, Sang-In Park, Sang-Myeong Lee, Kyung-Ah Hwang, Jun-Won Yun, Jae-Hwan Nam

**Affiliations:** ^1^Department of Medical and Biological Sciences, The Catholic University of Korea, Bucheon, South Korea; ^2^Department of Nuclear Medicine, Seoul National University Bundang Hospital, Seoul, South Korea; ^3^Preclinical Research Center, Seoul National University Bundang Hospital, Seoul, South Korea; ^4^Division of Biomedical Convergence, College of Biomedical Science, Kangwon National University, Chuncheon, South Korea; ^5^BK21 Program for Veterinary Science, Research Institute for Veterinary Science, College of Veterinary Medicine, Seoul National University, Seoul, South Korea; ^6^Laboratory of Avian Diseases, College of Veterinary Medicine, Seoul National University, Seoul, South Korea; ^7^Laboratory of Developmental Biology and Genomics, BK21 PLUS Program for Creative Veterinary Science Research, Research Institute for Veterinary Science, College of Veterinary Medicine, Seoul National University, Seoul, South Korea; ^8^Korea Mouse Phenotyping Center (KMPC), Seoul National University, Seoul, South Korea; ^9^Interdisciplinary Program for Bioinformatics, Program for Cancer Biology and BIO-MAX/N-Bio Institute, Seoul National University, Seoul, South Korea; ^10^Scripps Korea Antibody Institute, Chuncheon, South Korea; ^11^College of Veterinary Medicine, Chungbuk National University, Cheongju, South Korea; ^12^Department of Research and Development, SML Genetree, Seoul, South Korea; ^13^BK21 PLUS Program, The Catholic University of Korea, Bucheon, South Korea

**Keywords:** SARS-CoV-2, peptides, T cell responses, vaccine, CTL

## Abstract

The emergence of severe acute respiratory syndrome coronavirus 2 (SARS-CoV-2) variants that escape vaccine-induced neutralizing antibodies has indicated the importance of T cell responses against this virus. In this study, we highlight the SARS-CoV-2 epitopes that induce potent T cell responses and discuss whether T cell responses alone are adequate to confer protection against SARS-CoV-2 and describe the administration of 20 peptides with an RNA adjuvant in mice. The peptides have been synthesized based on SARS-CoV-2 spike and nucleocapsid protein sequences. Our study demonstrates that immunization with these peptides significantly increases the proportion of effector memory T cell population and interferon-γ (IFN-γ)-, interleukin-4 (IL-4)-, tumor necrosis factor-α (TNF-α)-, and granzyme B-producing T cells. Of these 20 peptides, four induce the generation of IFN-γ-producing T cells, elicit CD8^+^ T cell (CTL) responses in a dose-dependent manner, and induce cytotoxic T lymphocytes that eliminate peptide-pulsed target cells *in vivo*. Although it is not statistically significant, these peptide vaccines reduce viral titers in infected hamsters and alleviate pulmonary pathology in SARS-CoV-2-infected human ACE2 transgenic mice. These findings may aid the design of effective SARS-CoV-2 peptide vaccines, while providing insights into the role of T cells in SARS-CoV-2 infection.

## Introduction

The emergence of coronavirus disease 2019 (COVID-19) caused by severe acute respiratory syndrome coronavirus 2 (SARS-CoV-2), which is genetically linked to SARS-CoV, is a threat to global public health and economy. Owing to the rapid, global spread of SARS-CoV-2, the World Health Organization declared the COVID-19 outbreak a pandemic on March 11, 2020 (Valencia, 2020). As of February 22, 2021, more than 111 million individuals have been infected with SARS-CoV-2, and 2.46 million have succumbed to the disease. Therefore, several global institutes and companies have increased vaccine development in response to the COVID-19 pandemic.

Severe acute respiratory syndrome coronavirus 2 consists of approximately 30,000 nucleotides and encodes four structural proteins, namely the spike (S) glycoprotein, small envelope (E) glycoprotein, membrane (M) glycoprotein, and nucleocapsid (N) protein (Huang et al., 2020; Mousavizadeh and Ghasemi, 2020). The S protein is composed of two subunits, S1 and S2; the S1 subunit binds to the receptors of target cells through its receptor-binding domain (RBD), while S2 mediates the fusion of the viral membrane through conformational changes (Huang et al., 2020). Since the S protein is responsible for binding to host cells, most of the current SARS-CoV-2 vaccine candidates are based on the full-length S protein, as well as the S1 subunit and RBD (Krammer, 2020; Yang et al., 2020).

N proteins are highly immunogenic and abundantly expressed in many coronaviruses (Dutta et al., 2020). DNA vaccines encoding the SARS-CoV N protein have been shown to induce strong N-specific humoral and cellular immune responses in C57BL/6 mice and significantly reduce viral titers after an antigenic challenge (Kim et al., 2004). Meanwhile, Buchholz et al. (2004) have reported that N protein immunization does not induce neutralizing antibody responses and fails to confer protection against infection in the SARS-CoV hamster model. Although the SARS-CoV N protein can trigger both humoral and T cell responses in humans, further studies are warranted to clarify their protective effects for vaccine development.

Effective vaccines primarily depend on the induction of high levels of neutralizing antibodies; however, T cells also play critical roles in virus elimination and protection from viral infection. Particularly, cytotoxic CD8^+^ T cells (CTLs) directly eliminate virus-infected cells by producing cytotoxic granules, such as perforin, granzyme B, and interferon-γ (IFN-γ). Additionally, CD4^+^ T cells are essential for generating effective cellular and humoral immune responses by producing sufficient cytokines and by activating co-stimulation signaling (Swain et al., 2012). Although most studies conducted on SARS-CoV-2 vaccine development have focused on the elicitation of the humoral response (Dong et al., 2020), recent data have emphasized the importance of generating T cell-mediated responses as well. For instance, Thevarajan et al. (2020) have demonstrated that the levels of activated T cells increase at the time of SARS-CoV-2 clearance (Thevarajan et al., 2020). T cell receptor clonality is also higher in patients with mild symptoms compared to those with severe COVID-19 (Bacher et al., 2020).

The emergence of SARS-CoV-2 variants such as B.1.1.7 and B.1.351 that have been shown to escape vaccine-induced neutralizing antibodies (Fontanet et al., 2021) has shifted the focus to T cell vaccines (Hellerstein, 2020; Ledford, 2021; Tarke et al., 2021). Previously, SARS-CoV-specific memory T cells were found to exhibit reaction with membrane proteins, and the N protein was shown to persist for up to 11years after infection in recovered patients, whereas SARS-specific antibodies showed waned response after a few years (Ng et al., 2016). These results suggest that the induction of antigen-specific T cell responses may be helpful for the development of successful vaccines. However, it remains unclear whether T cell responses alone can exert protective effects against SARS-CoV-2 infection.

Therefore, the primary goal of the current study is to identify the SARS-CoV-2 epitopes that induce T cell responses and to assess the protective effects of this response. To this end, 20 peptides derived from the most conserved regions of SARS-CoV-2S and N proteins were synthesized and formulated with RNA adjuvants to enhance T cell immunogenicity *in vivo*. Furthermore, hamster, as well as human angiotensin-converting enzyme 2 (hACE2) transgenic (Tg) mouse models, was utilized to determine whether T cell responses induced by immunization with the RNA adjuvant + peptide mixture conferred protection against SARS-CoV-2 infection.

## Materials and Methods

### Mice and Hamsters

Six-week-old female C57BL/6 mice and golden Syrian hamsters were purchased from Samtako Biokorea (Osan, Republic of Korea) or Dae-Han Bio-Link (Chungbuk, Republic of Korea), while hACE2 mice (JAX Stock# 034860, B6. Cg-Tg(K18-ACE2)2 Prlmn/J/) were purchased from the Jackson Laboratory (ME, United States).

Animals were housed at the Catholic University of Korea under specific-pathogen-free conditions and a standard light cycle (12-h light/dark cycle). All animal experimental protocols were approved by the Institutional Animal Care and Use Committee of the Catholic University of Korea (approval number: CUK-IACUC-2020-015, BA-2008-301-071-01), whose animal facility is fully accredited by the Korean Association for Laboratory Animals (2018-027, August 24, 2018). All experimental procedures were conducted in accordance with the guidelines of the Institutional Animal Care and Use Committee of the Catholic University of Korea.

### Immunization

C57BL/6 WT mice were immunized *via* intramuscular injection (40μl) in the upper thigh twice at 2-week interval with the 20 SARS-CoV-2 peptides (each 10μg, a total of 200μg of peptides), four peptides (ST5, ST7, NT5, and NT6; 20μg per peptide, total 80μg) with or without the CUK2 RNA adjuvant (40μg), or only the CUK2 RNA adjuvant (40μg).

Human angiotensin-converting enzyme 2 transgenic mice were intramuscularly injected in the upper thigh twice with either the CUK2 RNA adjuvant or two peptides (ST5 and ST7; 20μg per peptide, total 40μg) or the CUK2 RNA adjuvant and four peptides (ST5, ST7, NT5, and NT6; 20μg per peptide, total 80μg) at 2-week interval.

The golden Syrian hamsters were immunized three times at 2-week interval with the SARS-CoV-2 peptides (15μg each at total peptides of 300μg) and CUK2 RNA adjuvant (60μg).

### SARS-CoV-2 Challenge

Human angiotensin-converting enzyme 2 transgenic mice were challenged with SARS-CoV-2 1week after the final immunization. Following the intraperitoneal administration of anesthesia using tiletamine+zolazepam (Zoletil 50; 30mg/kg)+xylazine (10mg/kg), each mouse was infected intranasally with 1.0×10^4^ plaque-forming units per 50 μl of SARS-CoV-2, and the survival rates were determined. All challenged mice were maintained in BSL-3 facilities at the Korea mouse phenotyping center of Seoul National University. All mouse experimental procedures were conducted in accordance with the guidelines of the Institutional Animal Care and Use Committee of Seoul National University (approval number: BA-2008-301-071-01).

Golden Syrian hamsters were challenged with SARS-CoV-2 1week after the final immunization. Following intraperitoneal administration of anesthesia using tiletamine+zolazepam (Zoletil 50; 30mg/kg)+xylazine (10mg/kg), each hamster was infected intranasally with 5.0×10^5^ plaque-forming units 50μl^−1^ of SARS-CoV-2. All challenged hamsters were maintained in BSL-3 facilities at the Korea Zoonosis Research Institute of Jeonbuk National University (JBNU). All hamster experimental procedures were conducted in accordance with the guidelines of the Institutional Animal Care and Use Committee of JBNU (approval number: JBNU2020-0155).

### Virus Culture and Titration

Severe acute respiratory syndrome coronavirus 2 virus (NCCP 43326), obtained from the National Culture Collection for Pathogens (NCCP) of the National Institute of Health, was propagated in Vero E6 cells (CRL-1586; Korean Collection for Type Cultures) in DMEM (Life Technologies, Carlsbad, CA, United States) supplemented with penicillin–streptomycin and 10% fetal bovine serum (Life Technologies) as per previously described methods (Kim et al., 2020). After determining the cytopathic effect of SAR-CoV-2 on day 3, virus titers were measured by using the median tissue culture infectious dose TCID_50_ and plaque assays.

### Prediction and Synthesis of T Cell Peptides (Epitopes) for SARS-CoV-2

The sequences of SARS-CoV-2 T cell epitope peptides were predicted by adopting *in silico* algorithms and methods described by previous studies on SARS-CoV. The SARS-CoV-2 strain Wuhan-Hu-1 (MN908947) was selected according to the information presented in various reviews and the NCBI databank. The surface glycoprotein (S; accession no. QHD43416.1) and nucleocapsid phosphoprotein (N; accession no. QHD43423.2) sequences were obtained from the NCBI GenBank database with the accession number in FASTA format. Coronavirus T cell epitopes were predicted by using the online epitope prediction server Immune Epitope Database (IEDB)^1^ and T cell epitope prediction tools NetMHCPan (PMID: 28978689). Epitopes selected from the prediction server had the lowest percentile rank and IC_50_ values according to the CONSENSUS method.

The peptide sequences used are as follows: S_277–297_(ASTEKSN), S_1132–1161_(KCYGVSPTKL), S_1309–1341_(NSNNLDSKVGG), S_1342–1371_(NYNYLYRLFR), S_1411–1449_(EIYQAGSTPCNGV), S_1459–1503_(NCYFPLQSYGFQPTN), S_1603–1659_(KNKCVNFNFNGLTGTGVLT), S_1924–1968_(VFQTRAGCLIGAEHV), S_1969–2016_(NNSYECDIPIGAGICA), N_40–72_(RITFGGPSDST), N_148–192_(ASWFTALTQHGKEDL), N_247–300_(QIGYYRRATRRIRGGDGK), N_328–372_(FYYLGTGPEAGLPYG), N_385–444_(GIIWVATEGALNTPKDHIGT), N_652–681_(ALALLLLDRL), N_715–762_(QQQQGQTVTKKSAAEA), N_934–963_(SAFFGMSRIG), N_988–1032_(WLTYTGAIKLDDKDP), N_1048–1092_(VILLNKHIDAYKTFP), and N_1219–1242_(LQQSMSSA).

All sequences were synthesized with a purity of 95% or more (Peptron, Daejeon, Republic of Korea). Each peptide was synthesized using a standard solid-phase peptide synthesis protocol. The Fmoc protecting group was removed by rocking in 20% piperidine in N, N-dimethylformamide (DMF) for 10min (twice), and coupling was performed using Fmoc amino acid (6eq), HOBT (6eq), HBTU (6eq), and DIPEA (12eq) in DMF for 2h. For each step, the resin was washed using DMF and methanol for two times each. When the desired sequence was complete, the crude peptide was cleaved from the resin using a mixture of TFA/EDT/Thioanisole/TIS/DW (90/2.5/2.5/2.5/2.5 Volume) for 2h. The solution was precipitated with cold ether, and pallets were obtained by centrifugation, collected, and then air-dried. The crude peptide was dissolved in deionized water (DW) and purified using reverse-phase high-performance liquid chromatography with a C18 reverse-phase column. Elution was performed with a water–acetonitrile linear gradient [10~75% (v/v) of acetonitrile] containing 0.1% (v/v) trifluoroacetic acid. The pure portion of the peptide was collected and lyophilized.

### *In vitro* Transcription and Purification of RNA Adjuvant

A DNA platform was designed using the encephalomyocarditis virus intergenic region internal ribosome entry site 5' UTR and 3' UTR with 50 adenylates at the 3' end (Ko et al., 2019) and termed as CUK2. DNA templates were linearized using Not I. *In vitro* transcription was performed using the EZ T7 High Yield *in vitro* Transcription Kit (Enzynomics, Daejeon, Republic of Korea), as per the manufacturer’s instructions. The transcription reactions contained 3μg of template DNA cut using Not I, 5x transcription buffer, 10x MgCl_2_, DTT (10mM final), rNTP (5mM final), 20x enhancer solution, T7 RNA polymerase, and nuclease-free water. The mixtures were incubated for 2h at 37°C. To remove the DNA, RNase-free DNase I (Promega, Wisconsin, United States) was added to the mixtures at 1unit/ug of template DNA and incubated for 30min at 37°C. For RNA purification, RNA was precipitated by adding 7.5M LiCl solution 0.5 volume and incubated for 30min at 4°C. After centrifugation for 15min at 13,000*g* and 4°C, the supernatant was discarded, and 600μl of 70% ethanol was added and centrifuged for 15min at 13,000*g* and 4°C for washing. The supernatant was discarded, and the pellet was resuspended with RNase-free water after air drying. The purity and concentration of DNA and RNA were evaluated using a NanoDrop-2000 spectrophotometer (Thermo Fisher Scientific, MA, United States).

### Viral Quantification

The viral RNA in the six lobes of the lungs was quantified using reverse transcription-quantitative PCR (RT-qPCR). Lung samples were homogenized using a Precellys Homogenizer (Bertin Instruments, Montigny-le-Bretonneux, France) in 10-fold (w/v) sterile phosphate-buffered saline (PBS; pH 7.4). Viral RNA was extracted from the supernatants of swab and tissue samples using a QIAamp Viral RNA Mini Kit (Qiagen, Germantown, MD, United States). Real-time one-step RT-PCR was performed using an Ezplex® SARS-CoV-2G Kit (SMLgenetree, South Korea) according to the manufacturer’s instructions. Viral RNA copy numbers from tissue samples were calculated using the standard control of the Accuplex™ SARS-CoV-2 Verification panel (Seracare, Milford, MA, United States).

### Enzyme-Linked Immunospot Assay

Enzyme-linked immunospot (ELISPOT) was used to detect IFN-γ- and interleukin-4 (IL-4)-producing T cells according to the manufacturers’ instructions (Mabtech, Stockholm, Sweden; R&D Systems. Minneapolis, MN, United States). Polyvinylidene fluoride plates were activated by treatment with 70% ethanol per well for 2min. After washing the plate five times with PBS, coating antibody (15μg/ml) was added and the samples were incubated overnight at 4°C. After washing the plate five times with PBS, RPMI media containing 10% fetal bovine serum was added, and the samples were incubated for 30min. After removing the media, the splenocytes from immunized mice collected at the end of the experiments were added at 5×10^5^ cells/well and stimulated with 5μg per well of peptide antigens for 48h at 37°C. The plates were then washed five times with PBS, and biotin-labelled anti-IFN-γ or IL-4 antibodies were added and incubated for 2h at 18 – 23°C. After washing, the plates were treated with diluted streptavidin-APL (1:1,000) and incubated for 1h at room temperature. After washing five times with PBS, stop solution (BCIP/NBT) was added and developed until distinct spots were observed. Reaction was terminated by washing in tap water. The plates were dried, and spots were counted using an ELISpot reader and analyzed using AID Elispot reader software 7.0 (AID GmbH, Strassberg, Germany).

### Flow Cytometry

For surface staining, the splenocytes and isolated immune cells from draining lymph nodes were stained with the following antibodies for 30min at 4°C: Fixable Viability Dye eFluor™ 520 (eBioscience, San Diego, CA, United States), anti-CD4 (clone GK1.5; eBioscience), anti-CD8 (clone 53–6.7; Invitrogen), anti-CD44 (clone IM7; eBioscience; BD Pharmingen), anti-CD62L (clone MEL-14; eBioscience), and anti-CD103 (clone 2E7; eBioscience). Cells were then fixed in 4% paraformaldehyde and analyzed using the Cytek Aurora flow cytometer (Cytek Biosciences, Fremont, CA, United States).

For transcription factor Ki-67 staining, the surface-stained splenocytes and isolated immune cells from draining lymph nodes were permeabilized using the Foxp3/Transcription Factor Staining Buffer Set (eBioscience) and stained with Ki-67 (clone SolA15; eBioscience) for 30min at 18 – 25°C. Cells were then analyzed using the Cytek Aurora flow cytometer.

For intracellular cytokine staining, the isolated splenocytes were re-stimulated with 5 μg per well SARS-CoV-2 peptide mixture at 37°C. Brefeldin A (Golgi-Plug; BD Biosciences, Franklin Lakes, NJ, United States) was added after 4h, and after incubation for another 12h, splenocytes were treated with anti-CD16/CD32 (eBioscience) for 30min at 4°C and then stained with the following antibodies for 30min at 4°C: Fixable Viability Dye eFluor™ 520, anti-CD4 (clone GK1.5), and anti-CD8 (clone 53–6.7). The stained cells were permeabilized using the Cytofix/Cytoperm kit (eBioscience) and then stained with anti-IFN-γ (clone XMG1.2; eBioscience), anti-tumor necrosis factor-α (TNF-α; clone MP6-XT22; eBioscience), anti-granzyme B (QA16A02; BioLegend, San Diego, CA, United States), and anti-perforin (S16009A; BioLegend) antibodies. Cells were analyzed using the Cytek Aurora flow cytometer.

### Cytokine Enzyme-Linked Immunosorbent Assay

To measure cytokine levels in splenocyte culture supernatants, mouse splenocytes were collected and isolated from immunized mice. Splenocytes were seeded at a density of 5×10^5^ cells per well (96-well plate). To re-stimulate the splenocytes, 5μg per well of the SARS-CoV-2 peptide mixture was added to the culture medium for 4days, after which the medium was assessed using ELISA to determine the concentrations of IFN-γ, IL-2, IL-6, and TNF-α (Invitrogen; Thermo Fisher Scientific, Waltham, MA, United States), according to the manufacturer’s instructions. Further, the 96-well plates were coated with the capture antibodies of IFN-γ, IL-2, IL-6, and TNF-α and incubated overnight at 4°C. After incubation, the plates were washed three times with 0.05% Tween-20 in PBS and blocked with 1x diluent for 1h at room temperature. After washing the plates, 50μl of culture supernatants was added and incubated for 2h at room temperature. The plates were washed again, treated with the diluted detection antibodies of IFN-γ, IL-2, IL-6, and TNF-α, and incubated for 1h. After washing the plates, diluted avidin-HRP or streptavidin-HRP was added and the samples were incubated for 30min. After washing five times, 100μl of 1x TMB solution was added and incubated for 15min, and 2N H_2_SO_4_ was used to terminate the reaction. The optical density values were measured at 450nm using a GloMax Explorer Multimode Microplate Reader (Promega, WI, United States). Cytokine concentrations were calculated according to standard curves, and the obtained results are shown as IFN-γ, IL-2, IL-6, and TNF-α pg per ml of the supernatant.

### *In vivo* CTL Assay

C57BL/6 mice were immunized with peptide mixture or each of the four peptides (ST5, ST7, NT5, and NT6) or S1 peptides (ST5+ST7) or N peptides (NT5+NT6) together with CUK2 RNA adjuvant, twice at a 2-week interval. Donor splenocytes from unimmunized mice were obtained, then subjected to washing steps, and divided into two groups based on cell populations. One group was pulsed with 10μg/ml SARS-CoV-2 peptide for 30min at 37°C, subjected to washing steps using PBS, and labeled with a high concentration (2.5μM) of carboxyfluorescein succinimidyl ester (CFSE). Another control group was labeled with a low concentration of CFSE (0.25μM). Cells (5×10^6^) of each population were mixed with 200ml of PBS and intravenously injected into each peptide + CUK2 RNA adjuvant immunized mouse 7days after second immunization. Specific *in vivo* cytotoxicity was determined by collecting spleens from recipient mice 24h after injection to determine the number of cells in each target cell population *via* flow cytometry. The ratio between the percentage of unpulsed vs pulsed peptides (CFSE_low_/CFSE_high_) was quantitatively assessed for determining cytotoxicity. Further controls included naive- and PBS-immunized recipient mice. The percentage of specific lysis in control vs. immunized mice was calculated according to the following equation:

% Specific lysis=100−[100×(%CFSE_high_ immunized mouse/%CFSE_low_ immunized mouse)/(%CFSE_high_ naive mouse/%CFSE_low_ naive mouse)].

### Histological Analysis

Sectioned liver tissue samples obtained from mice were submerged in 10% (v/v) neutral-buffered formalin, dehydrated, paraffin-embedded, and sectioned at a thickness of 3μm for conducting histological analysis. Representative histopathological images were obtained and evaluated using Aperio ImageScope (version 12.3; Leica Biosystems, Wetzlar, Germany). The severity of the histopathological changes was determined using a five-point scoring system as follows: 0, no abnormality detected; 1, minimal; 2, mild; 3, moderate; 4, moderately severe; and 5, severe, and distribution was recorded as focal, multifocal, and diffused. Recruitment of inflammatory cells to the liver (blood vessel or hepatic parenchymal region) and morphological alterations (sinusoidal dilatation, necrosis, and ballooning degeneration) of the tissue were assessed after the performance of hematoxylin and eosin staining and visualization under a light microscope.

### Statistical Analysis

All values are expressed as mean±SD. One-way ANOVA was used to assess significant differences among treatment groups; if significant deviations from variance homogeneity were detected using the Levene test, then the nonparametric Kruskal–Wallis H test was performed. Student’s *t* test was used to assess significant differences between two groups; if significant deviations from variance homogeneity were detected using the Levene test, then the nonparametric Mann–Whitney U test was performed. Statistical analyses were conducted using SPSS for Windows (release 14.0K, IBM, Armonk, NY) or GraphPad Prism (GraphPad Software Inc., La Jolla, CA). Differences were considered significant at values of *p* ≤0.05.

## Results

### *In silico* Prediction and Selection of SARS-CoV-2 Peptides

**Figure 1:**
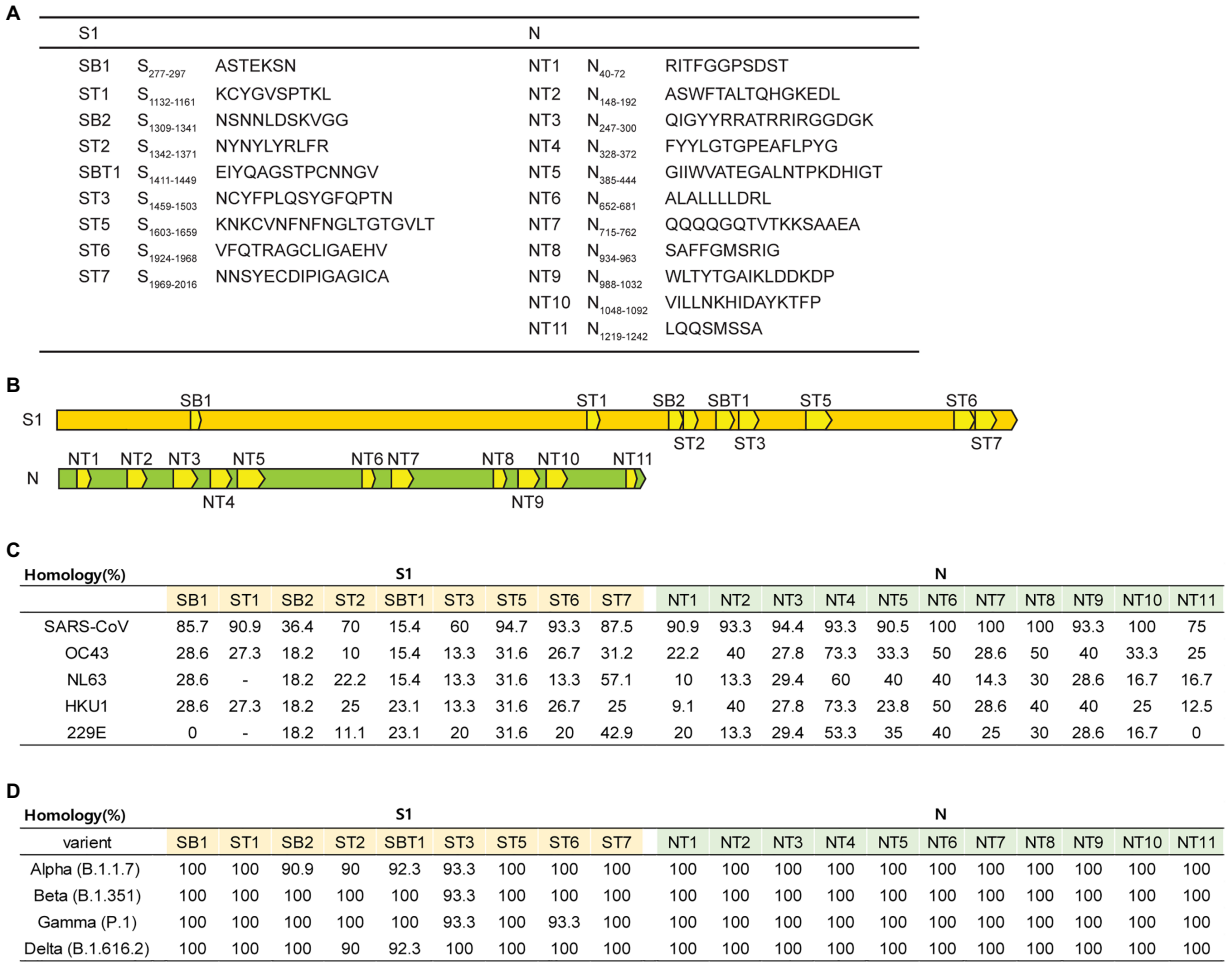
Overview of the 20 peptides derived from severe acute respiratory syndrome coronavirus 2 (SARS-CoV-2) S and N proteins. **(A)** List of 20 peptide sequences. **(B)** Location of the 20 designed peptides in the S and N proteins. **(C,D)** Homology percentage of each peptide to other coronaviruses [SARS-CoV, OC43, NL63, HKU1, 229E, and SARS-CoV-2 variants (α, β, γ, and δ)].

To develop the SARS-CoV-2 peptide vaccine, the T and B cell epitopes of SARS-CoV-2 peptides were predicted using *in silico* algorithms and the IEDB with the reference virus strain Wuhan-Hu-1 (MN908947) as described in Material and Methods. Briefly, 9 S peptide sequences with a conserved S1 region in SARS-CoV-1(AAP 41037.1) and 11 N peptide sequences with conserved region in N protein of SARS-CoV-1(AAP 41047.1) were selected (Figures 1A,B). We also analyzed the homology of each peptide to other coronaviruses and SARS-CoV-2 variants (α, β, γ, and δ). As shown in Figure 1C, 20 peptides showed the highest homology with SARS-CoV of S1 and N protein (36.4–100%), and most of these selected peptides also have homology with other seasonal coronaviruses but lesser extent compared to those of SARS-CoV (Figure 1C). Fifteen peptides except SB2, ST2, SBT1, ST3, and ST6 showed 100% homology to SARS-CoV-2 variants (α, β, γ, and δ; Figure 1D). Since we aimed to test the immunogenicity of these peptides in mice, 20 peptides predicted to bind H-2K^b^, H-2D^d^, H-2D^b^, H-2K^d^ [major histocompatibility complex (MHC) class I], and H-2IE^d^ and H-2-IA^b^ were selected. All peptides were synthesized as described in Material and Methods.

### Induction of T Cell Response After Immunization With the SARS-CoV-2-Derived 20-Peptide + RNA Adjuvant Mixture

**Figure 2:**
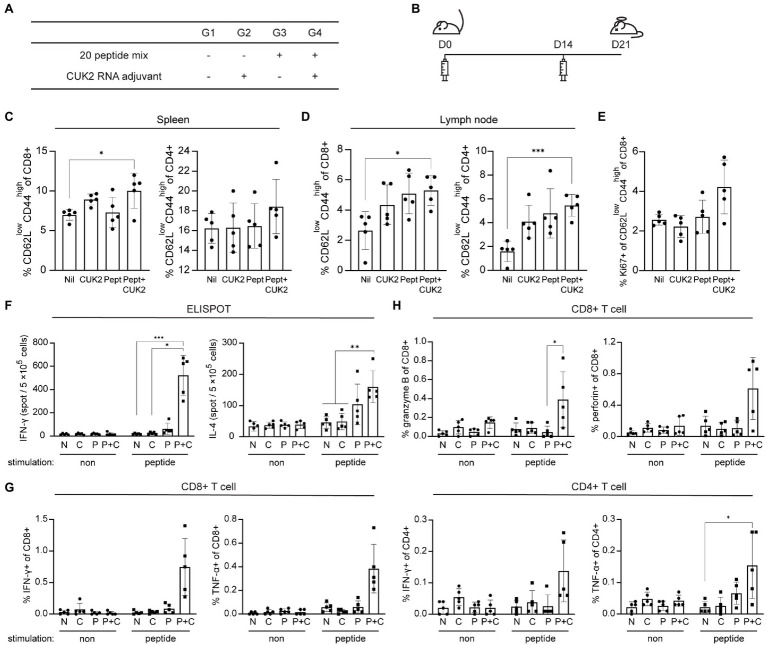
Analysis of T cell activation after immunization with the SARS-CoV-2 20-peptide mixture and CUK2 RNA adjuvant. **(A)** Overview of the experimental groups. **(B)** Immunization schedule of mice. C57BL/6 mice were immunized twice at 2-week intervals with the 20 SARS-CoV-2 peptides mixture + CUK2 RNA adjuvant and killed 1week after the final immunization. **(C,D)** The percentages of effector memory type of CD4+ and CD8^+^ T cells (CD62L^low^ CD44^high^) in the spleen and lymph nodes were analyzed using flow cytometry. **(E)** The Ki-67+ population in CD8^+^ effector memory T cells in the spleen was assessed using flow cytometry. **(F)** The numbers of 20 peptide-specific interferon-γ (IFN-γ)- and interleukin (IL)-4-producing cells in splenocytes were measured using enzyme-linked immunospot (ELISPOT; *n*=5 mice). **(G)** IFN-γ- and tumor necrosis factor-α (TNF-α)-producing CD8^+^ and CD4^+^ T cells in the spleen were examined using flow cytometry. **(H)** Granzyme B- and perforin-producing CD8^+^ T cells in the spleen were analyzed using flow cytometry. Data represent mean±SD. ^*^p≤0.05; ^**^p≤0.01; and ^***^p≤0.005.

Unlike Alum, as an adjuvant, RNA derived from the internal ribosome entry site in the 5' untranslated region of the encephalomyocarditis virus (designated CUK2) was used as it was previously determined that peptides or proteins formulated with this RNA adjuvant could induce antigen-specific T cell activation (Nam et al., 2020). To assess the induction of T cell immune responses *in vivo*, C57BL/6 mice were immunized intramuscularly with the 20-peptide mixture, with or without the CUK2 RNA adjuvant (Figures 2A,B). As indicated in Figures 2C,D, immunization peptides with CUK2 significantly increased the proportion of CD44^hi^CD62L^low^ effector memory CD4^+^ and CD8^+^ T cells (T_EM_ cells) in the spleen and CD8^+^ T_EM_ cells in the draining lymph nodes compared with the non-immunized group (G1). Although only peptides (G3) or CUK2 RNA adjuvant (G2) alone seemed to increase the proportion of effector memory type T cells in draining lymph nodes, the difference was not significant (Figure 2D). Moreover, the expression of Ki-67, a marker of recently proliferated cells, seems to be increased in CD8^+^ T_EM_ cells from mice immunized with the CUK2 RNA adjuvant + peptide mixture without statistical significance (G4; Figure 2E).

To assess whether the RNA adjuvant+20-peptide mixture affected cytokine production of T cells and not T cell phenotype and subsets, the number of cytokine-producing cells was quantified by ELISPOT and flow cytometry. A marked increase in the number of IFN-γ- and IL-4-producing cells was observed in the spleen along with an increase in the percentage of IFN-γ- and TNF-α-producing CD4^+^ and CD8^+^ T cells in CUK2 RNA adjuvant + peptide mixture immunized mice (G4; Figures 2F,G). We observed similar increase in IFN-γ- and TNF-α-producing CD4^+^ and CD8^+^ T cells in CUK2 RNA adjuvant + peptide mixture immunized Balb/c mice (data not shown). Similarly, splenocyte culture supernatants from the RNA adjuvant+peptide mixture-immunized group (G4) showed significantly increased IFN-γ, TNF-α, IL-2, and IL-6 levels (Supplementary Figure S1). Only the peptide mixture or RNA adjuvant alone did not significantly increase IFN-γ or TNF-α levels. Although IL-6 production increased in the peptide-immunized group (G3), the extent of increase was higher in the RNA adjuvant + peptide mixture-immunized group (G4; Supplementary Figure S1). To further evaluate the cytotoxic activity of T cells, granzyme B production by CD8^+^ T cells was assessed and found to be significantly increased in the RNA adjuvant+peptide mixture-immunized group (G4; Figure 2H). Thus, the results indicate that the peptide mixture could induce T cell immune responses *in vivo* when combined with an RNA adjuvant.

### Identification of Potent T Cell Epitopes of SARS-CoV-2 in Mice

**Figure 3:**
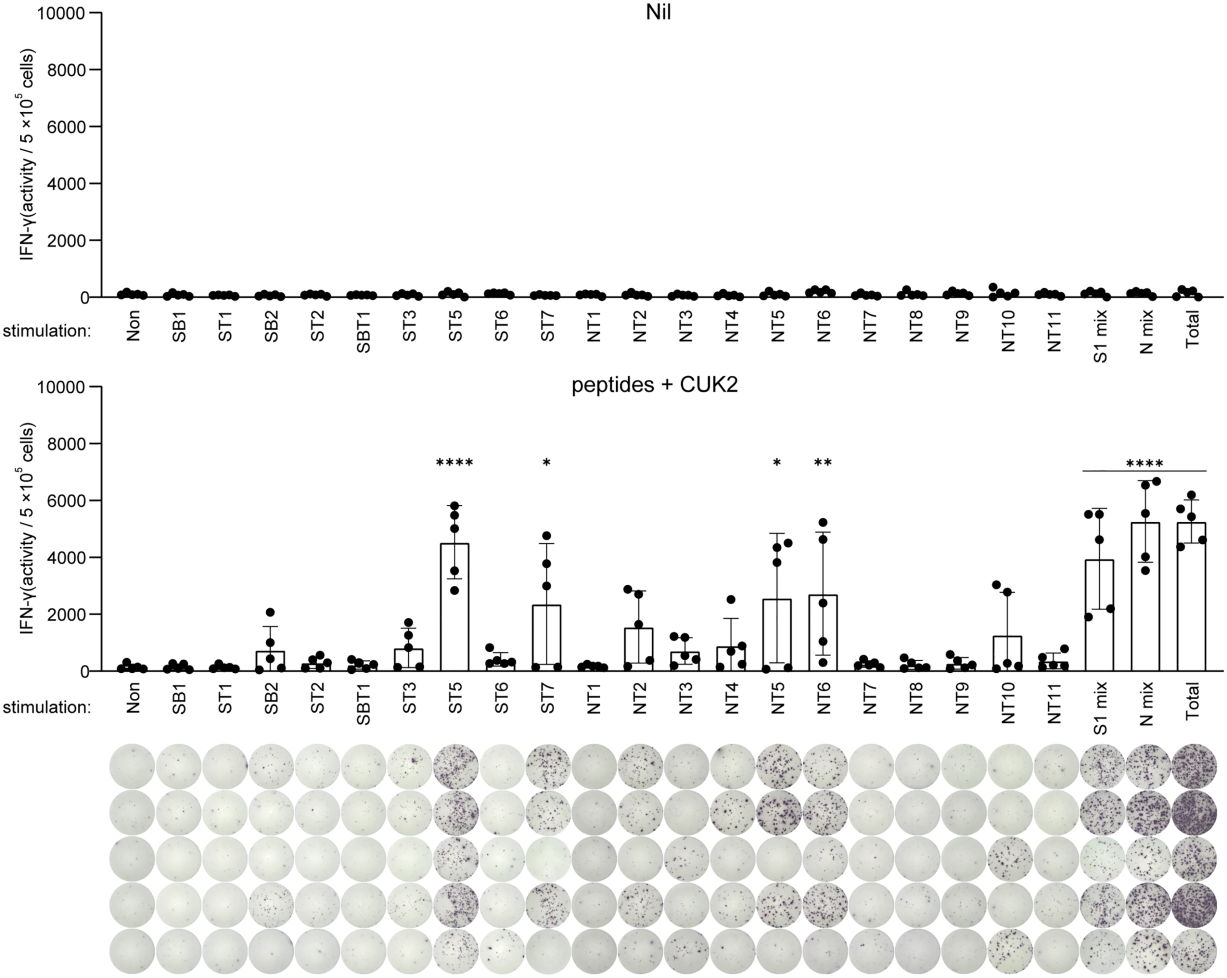
Analysis of IFN-γ-producing T cells after stimulation with each single peptide. C57BL/6 mice were immunized twice at 2-week intervals with the 20 SARS-CoV-2 peptides mixture+CUK2 RNA adjuvant (lower). The nil group included unimmunized C57BL/6 mice as a negative control (upper). Each peptide-specific IFN-γ-producing T cells in the spleen were quantified using ELISPOT. Data represent mean±SD. ^*^p≤0.05; ^**^p≤0.01; and ^****^p≤0.001.

Next, to identify the most potent T cell epitopes among the 20 peptides, splenocytes from immunized or unimmunized mice were stimulated with each peptide, and the number of IFN-γ-producing cells was determined by ELISTPOT (Figure 3). ST5 and ST7 peptides of the S1 subunit, as well as NT5 and NT6 peptides of the N protein, were found to strongly induce IFN-γ production (Figure 3). These selected ST5, ST7, NT5, and NT6 were almost identical to SARS-CoV and showed 100% homology to SARS-CoV-2 variants (α, β, γ, and δ; Figure 1D). Therefore, subsequent experiments were conducted with the four peptides ST5, ST7, NT5, and NT6.

### ST5, ST7, NT5, and NT6 Peptides Induce CD8^+^ T Cell Responses

**Figure 4:**
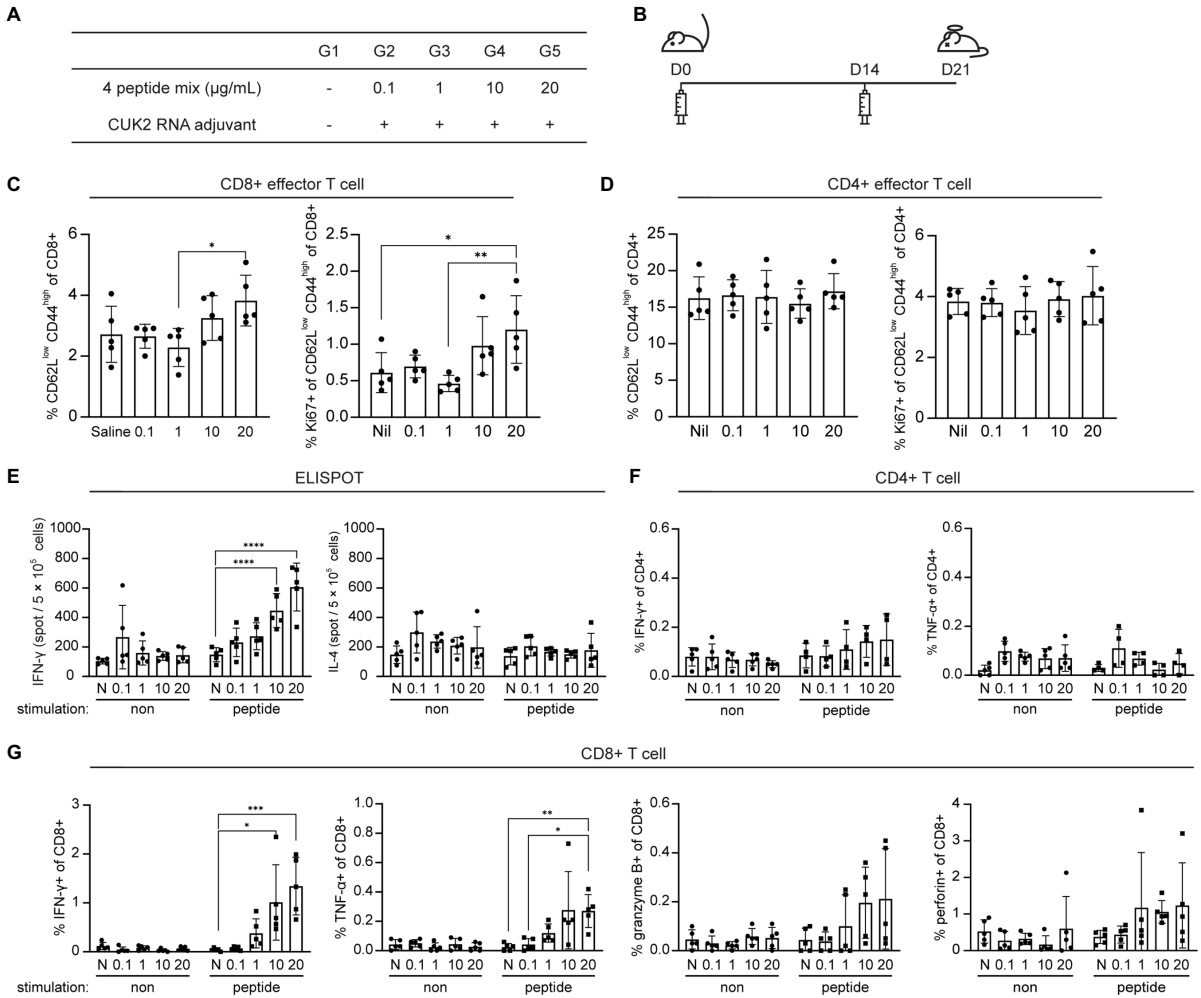
Analysis of T cell activation after immunization with the four SARS-CoV-2 peptides and CUK2 RNA adjuvant. **(A)** Overview of the experimental groups. **(B)** Schedule of mice immunization. C57BL/6 mice were immunized twice at 2-week intervals with the four SARS-CoV-2 peptides mixture+ CUK2 RNA adjuvant and killed 1week after the final immunization. **(C)** Percentages of CD62L^low^ CD44^high^ T_EM_ cells in the splenic CD8+ T cells and Ki67-positive cells in CD8^+^CD62L^low^ CD44^high^ T_EM_ CD8^+^ T cells were analyzed using flow cytometry. **(D)** Percentages of CD62L^low^ CD44^high^ T_EM_ cells in the splenic CD4^+^ T cells and Ki67-positive cells in CD8^+^CD62L^low^ CD44^high^ T_EM_ CD4^+^ T cells were analyzed using flow cytometry. **(E)** The number of four peptide mixture-specific IFN-γ- and IL-4-producing cells was quantified using ELISPOT. **(F)** IFN-γ- and TNF-α-producing cells in CD4^+^ T cells in response to four peptide mixtures were examined using flow cytometry. **(G)** IFN-γ- and TNF-α-producing cells in CD8^+^ T cells in response to four peptide mixtures were examined using flow cytometry. Data represent mean±SD. ^*^p≤0.05; ^**^p≤0.01; ^***^p≤0.005; and ^****^p≤0.001.

To determine the appropriate peptide dose of selected peptides, mice were immunized with 0.1, 1, 10, and 20μg of each peptide (ST5, ST7, NT5, and NT6) in the presence of the CUK2 RNA adjuvant (Figures 4A,B). A significant increase in the levels of Ki-67 expression in effector CD8^+^ T cells, but not CD4^+^ T cells, was observed only at a peptide concentration of 20μg (G5; Figures 4C,D). Notably, unlike the 20-peptide mixture, these four peptides did not induce CD4^+^ T cell responses (Figures 4D,F). Furthermore, although the CUK2 RNA adjuvant + four peptide mixture did not increase the number of IL-4-producing cells, it increased the percentage of IFN-γ- and TNF-α-producing CD8^+^ T cells in a dose-dependent manner (Figures 4E,G). Therefore, the results indicate that ST5, ST7, NT5, and NT6 are CD8^+^ T cell epitopes.

### ST5, ST7, NT5, and NT6 Induce *in vivo* CTL Responses

**Figure 5:**
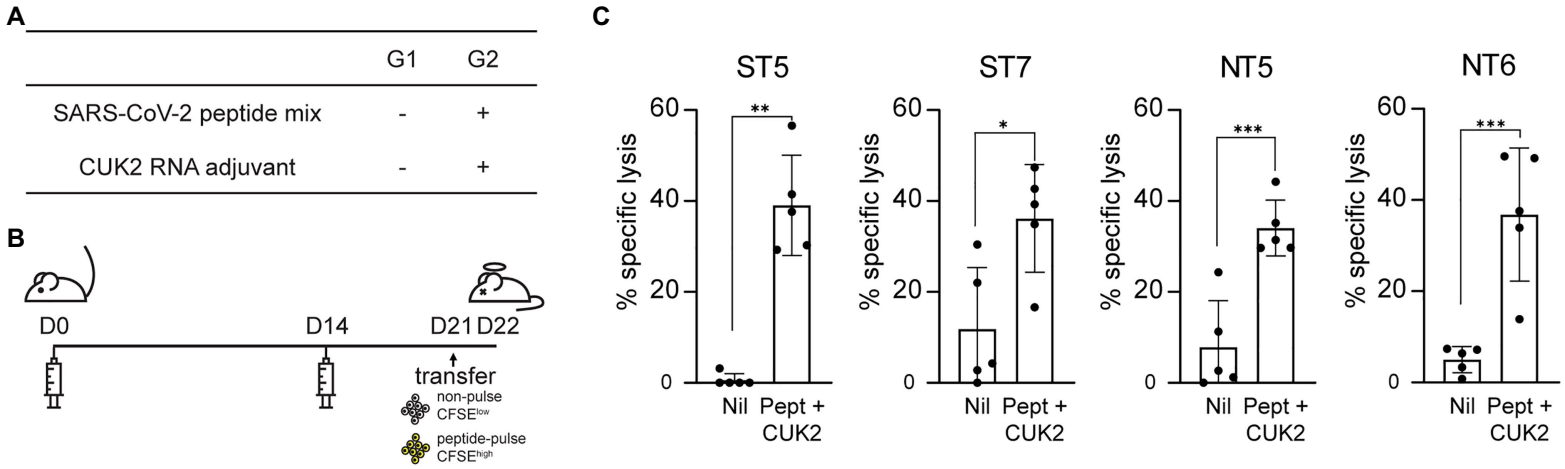
Severe acute respiratory syndrome coronavirus 2 peptide-specific CTL responses *in vivo*. **(A)** Overview of the experimental groups. **(B)** Schedule of mice immunization. C57BL/6 mice were immunized twice at 2-week intervals with each SARS-CoV-2 peptides mixture+ CUK2 RNA adjuvant and killed 1week after the final immunization. Carboxyfluorescein succinimidyl ester (CFSE), a fluorescent cell staining dye, was used. **(C)** Quantification of specific lytic responses from flow cytometric analysis. Data represent mean±SD. ^*^p≤0.05; ^**^p≤0.01; and ^***^p≤0.005.

Considering the four selected peptides increased CD8^+^ T cell expression of perforin and granzyme B (Figure 4G), which play critical roles in the direct elimination of virus-infected or cancer cells, it was subsequently determined whether these peptides also induced CTL responses *in vivo* using CTL assay. Mice were immunized with each of selected peptides as shown in Figures 5A,B. Mice immunized with each of the four peptides (ST5, ST7, NT5, or NT6)+CUK2 RNA adjuvant showed peptide-specific lytic responses, with the percentage of specific lysis ranging from 30 to 40% (Figure 5C). Mice immunized with the 20-peptide mixture+CUK2 RNA adjuvant also showed peptide-specific lysis; however, the lysis occurred to a lesser extent than that observed in the single peptide-immunized groups (Supplementary Figures S2A–C). Moreover, immunization with two S peptides (ST5 and ST7)+CUK2 RNA adjuvant or the two N peptides (NT5 and NT6)+CUK2 RNA adjuvant induced an *in vivo* CTL response (Supplementary Figure S2D).

### Evaluation of the Protective Effect of SARS-CoV-2 Peptides *in vivo*

**Figure 6:**
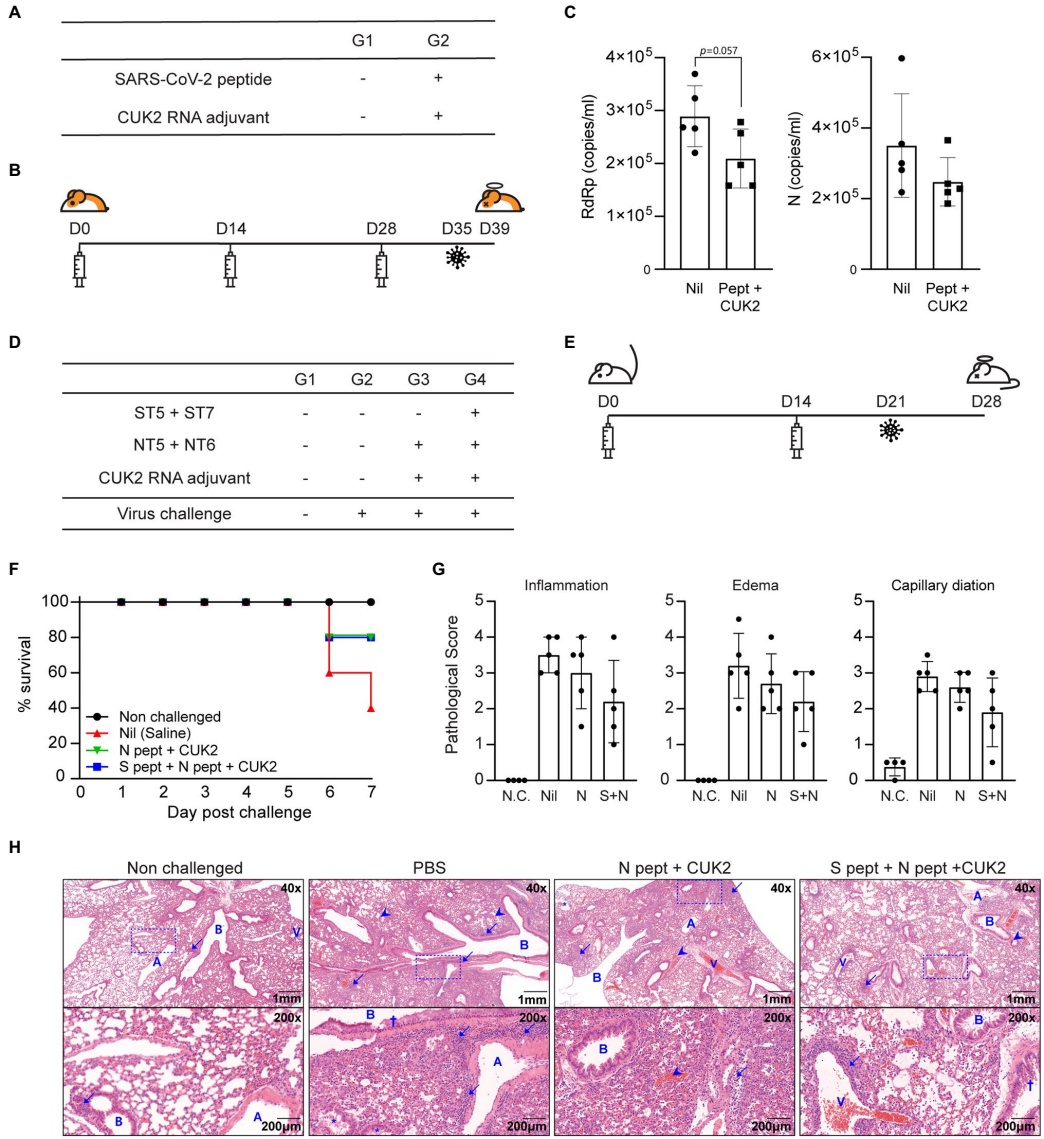
Immunization with SARS-CoV-2 peptides partially reduced the viral titer in hamsters and conferred protection to hACE2 mice against SARS-CoV-2 infection. **(A)** Overview of the experimental groups. **(B)** Immunization and virus challenge schedule. **(C)** Real-time PCR of viral genomic RNA in the lungs. Data are presented as mean±SD. **(D)** Overview of the experimental groups. **(E)** Immunization and virus challenge schedule. **(F)** Survival rates of the hACE2 Tg mice after SARS-CoV-2 challenge. **(G)** Pathological scores of lungs 7days after challenge. **(H)** Representative histological images of hematoxylin and eosin-stained lung tissue sections. A, pulmonary artery; B, bronchus or bronchi; and V, pulmonary vein. Arrows indicate inflammatory cells (neutrophils and lymphocytes), arrowheads indicate hemorrhage, and the crosses indicate mucus. Data represent mean±SD.

Considering that the RNA adjuvant and peptide mixture induced T cell responses, it was determined whether peptide-induced T cell responses conferred protection against the SARS-CoV-2 challenge. Golden Syrian hamsters were immunized three times using 15μg each of the 20 peptides (total 300μg) and 60μg of the CUK2 RNA adjuvant at 2-week interval and subsequently challenged with SARS-CoV-2 1week after conduction of the last immunization (Figures 6A,B). Although not statistically significantly, the copy numbers of the RNA-dependent RNA polymerase (RdRp) and N protein decreased in the CUK2 RNA adjuvant+peptide mixture-immunized group compared with those in the control group at 4days post challenge (Figure 6C).

Next, the protective effect of the selected peptides in a hACE2 Tg mouse model was assessed. hACE2 Tg mice were immunized twice with the CUK2 RNA adjuvant and two peptides (ST5 and ST7; 20μg per peptide, total 40μg) or four peptides (ST5, ST7, NT5, and NT6; 20μg per peptide, total 80μg) at 2-week interval and then challenged with SARS-CoV-2 1week after the conduction of the last immunization (Figures 6D,E). Both groups showed an improved survival rate (80%) 7days after the challenge compared with that of the control mice (40%; Figure 6F) but was not significant. Furthermore, the pathological scores (inflammation, edema, and capillary dilation) seem to be reduced in the RNA adjuvant+four peptide group compared with the control without statistical significance; particularly, in four out of five mice, a reduction in inflammatory lesions was observed, along with changes in the edematous response patterns around the respiratory tract compared with the control group (Figures 6G,H).

### Binding Prediction of the SARS-CoV-2-Derived Peptides to Human Leukocyte Antigen

**Figure 7:**
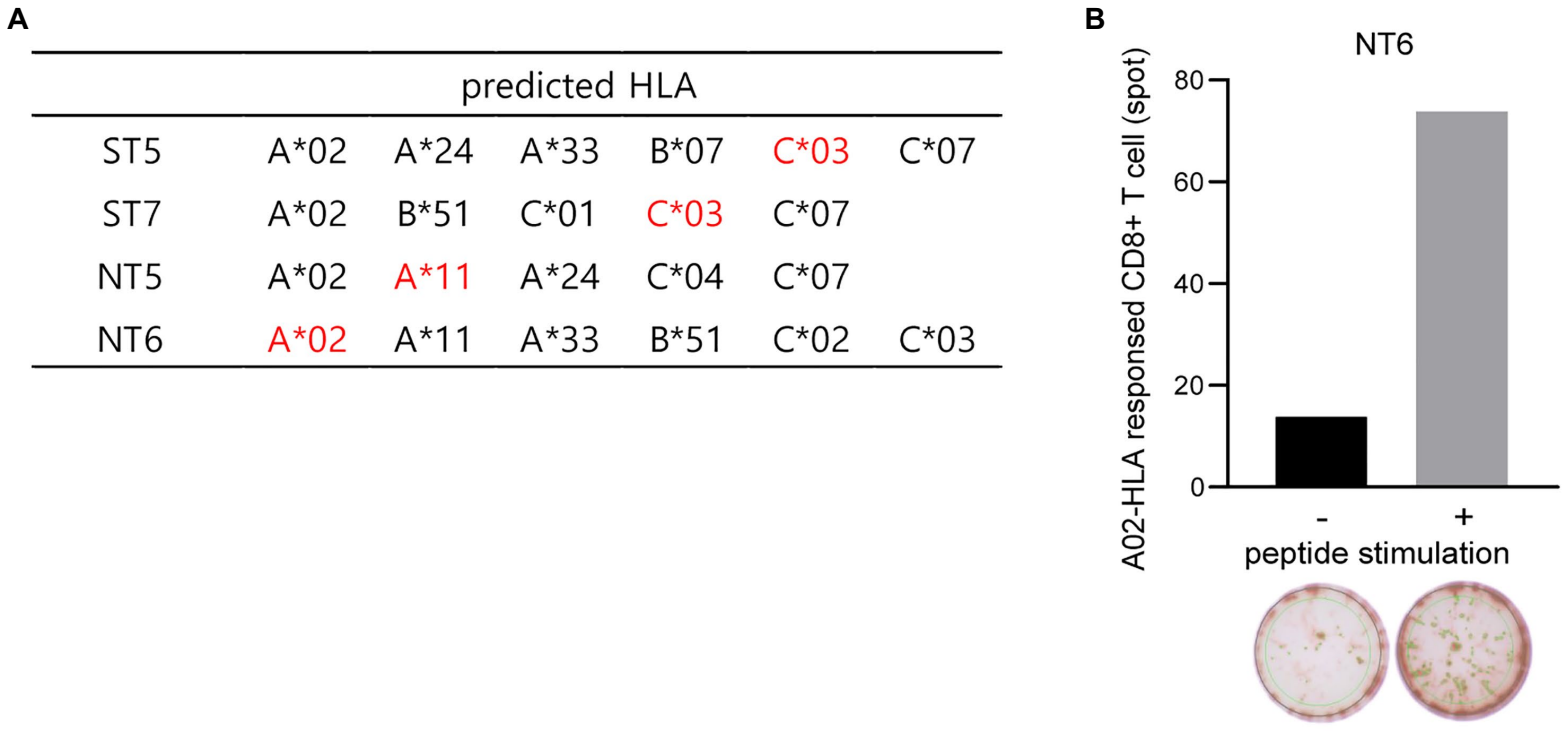
Binding prediction of the SARS-CoV-2-derived peptides to human HLA and *in vitro* CTL response in human PBMCs. **(A)** Human HLA predicted to bind to the selected four peptides. Red indicates HLA predicted to bind mainly to each peptide. **(B)** Detection of the NT6 peptide-specific CTL in human HLA-A*02-type T cells *via in vitro* induction using a peptide-specific CTL assay.

To determine whether these 20 selected peptides interacted with the MHC class I and induced CTL responses in humans, the binding affinity between human leukocyte antigen (HLA) class I and the 20 peptides, which would strongly contribute to the immunogenic effect of CTLs, was predicted using NetMHCPan (PMID: 28978689). Considering the binding affinity of peptides, peptides with low percentile rank (≤ 10) were selected as epitopes (data not shown). All four peptides were predicted to bind to several human HLA class I, such as HLA-A*02, A*024, A*11, C*03, and C*07, as indicated in Figure 7A, and NT6 is predicted bind to HLA-A*02-type donors with high affinity. Thus, we experimentally determined whether NT6 could induce CD8^+^ T cell responses in HLA-A*02-type donors. CD8^+^ T cells were co-cultured and subjected to expansion with autologous dendritic cells (DCs) pulsed with or without NT6. The level of IFN-γ produced by CD8^+^ T cells co-cultured with the NT6-pulsed DCs was five times greater than that produced by CD8^+^ T cells cultured without NT6 (Figure 7B). Thus, this result indicates that HLA-A*02-loaded NT6 could activate CD8^+^ T cells (Figure 7B).

### Safety Profile of the RNA Adjuvant + Peptide Mixture

To determine whether the CUK2 RNA adjuvant + peptide mixture was safe for usage, *in vivo* toxicity testing was performed (Supplementary Figures S3A,B). Mice were immunized twice with 200μg each of the 20 SARS-CoV-2 peptides (total 4mg), with or without the CUK2 RNA adjuvant, or CUK2 RNA adjuvant alone at 2-week intervals and killed 1week after the second immunization. Although alanine aminotransferase and aspartate aminotransferase serum levels slightly increased in the CUK2 RNA adjuvant + peptides and peptides alone groups, respectively, they remained within the normal ranges. All other toxicity indices, such as total bilirubin, creatinine, and albumin, did not significantly change and did not deviate from the normal ranges (Supplementary Figure S3C). Moreover, liver tissue samples obtained from immunized mice did not exhibit any noticeable morphological changes due to toxicity (Supplementary Figures S5D,E).

## Discussion

Traditionally, analyses using neutralizing antibodies have been regarded as the gold standard for evaluating vaccine efficacy (Kwong et al., 2011; Klasse, 2014). However, T cell response remains important. Owing to the emergence of SARS-CoV-2 variants that resist neutralizing antibodies elicited by prior infection or vaccination (Weisblum et al., 2020; Fontanet et al., 2021), there is an increasing interest in the development of vaccines that induce T cell responses. In addition to expanding and improving the effector function of immune cells, T cells also eliminate virus-infected cells (Pennock et al., 2013). In patients with COVID-19, an increased clonality of expanded CD8^+^ T cells was observed in patients with mild COVID-19 than in those with severe COVID-19 (Chen and John Wherry, 2020; Sekine et al., 2020). Furthermore, patients with severe COVID-19 exhibit not only lower numbers of cytotoxic CD8^+^ T cells but also a decreased capacity to produce IFN-γ and perforin (Mazzoni et al., 2020). These results suggest the association of T cells with the clearance of virus-infected cells. In contrast, it has been reported that immunization with a vaccine that selectively induces CD4^+^ T cell response results in increased occurrence of inflammation and mortality after chronic lymphocytic choriomeningitis virus infection (Penaloza-MacMaster et al., 2015). Additionally, CD8^+^ T cell-mediated immunopathology is observed in respiratory syncytial virus and influenza virus infection models (Schmidt et al., 2018). Therefore, establishment of a balance between immune protection and immune pathology by T cells is critical for the design of successful vaccines.

In this study, 20 peptides associated with the S and N proteins were selected that are highly conserved among SARS-CoV and SARS-CoV-2 and were evaluated for their T cell immunogenicity. To boost the low immunogenicity of peptides, CUK2 was used as an RNA adjuvant since we already have shown that it induces Th1 responses even in aged mice. Although T cell epitopes of SARS-CoV-2 have been predicted in several studies *via in silico* analysis (Dagur and Dhakar, 2020; Kiyotani et al., 2020; Wang et al., 2020), they have not been confirmed *in vivo*. In the present study, ST5, ST7, NT5, and NT6 peptides were found to induce T cell-mediated responses *in vivo*. Mice immunized with a mixture of these peptides and the CUK2 RNA adjuvant showed increased levels of IFN-γ-producing T cells and an increased frequency of proliferating T_EM_ cells. Furthermore, the amino acid sequences of ST5, ST7, NT5, and NT6 peptides were determined to be potent epitopes for cytotoxic CD8^+^ T cells as mice immunized with these peptides and the CUK2 RNA adjuvant showed robust CTL responses *in vivo*. However, the *in vivo* efficacy of select peptides may differ in humans due to the inherent differences in the MHC molecules of humans and mice. Nevertheless, NT6 peptides were confirmed to be recognized by CD8^+^ T cells from HLA-A*02 donors (data not shown), thus warranting further peptide screening with human T cells for application in clinical trials.

Although hamsters and hACE2 Tg mice immunized with the RNA adjuvant + peptide mixture showed slight reduction in viral titers and in the infliction of virus-induced injury in the lungs after SARS-CoV-2 challenge, it was not statistically significant. This indicates that the induced T cell responses induced by peptides are beneficial for the clearance of SARS-CoV-2 but are not sufficient for completely eliminating the virus. Therefore, the use of other adjuvants or strategies that optimize peptide-induced immune responses may provide a more effective protection. Additionally, our results demonstrated that T cell responses induced by S and N peptides did not completely attenuate SARS-CoV-2-induced pathology, indicating that stimulation of neutralizing antibody production is a key requirement for obtaining complete protection from SARS-CoV-2 infection. Nevertheless, we identified the S and N protein-associated peptides of SARS-CoV-2 that were capable of inducing T cell responses *in vivo* and provided a proof of concept of the development of T cell peptide vaccines against SARS-CoV-2. Based on these results, a SARS-CoV-2 vaccine consisting of SARS-CoV-2-derived proteins and peptides, with an aim to maximize both humoral and T cell responses, is presently under development.

## Data Availability Statement

The original contributions presented in the study are included in the article/Supplementary Material, further inquiries can be directed to the corresponding author.

## Ethics Statement

The animal study was reviewed and approved by Institutional Animal Care and Use Committee of the Catholic University of Korea.

## Author Contributions

J-HN conceived and supervised the research, designed the experiments, and edited the manuscript. Y-SL, S-HH, H-JP, H-YL, J-YH, SK, JP, K-SC, JS, S-IP, S-ML, K-AH, and J-WY performed data acquisition. S-HH, Y-SL, and H-JP provided assistance in drafting of the manuscript. All authors contributed to the article and approved the submitted version.

## Funding

work was supported by the Research of Korea Centers for Disease Control and Prevention (grant number 2020-ER5303-00), the Ministry of Food and Drug Safety in 2020 (grant number 20172MFDS290), the Catholic University of Korea, Research Fund, 2021, the Korea Mouse Phenotyping Project (grant number 2020M3A9I2109027) of the National Research Foundation (NRF) funded by the Ministry of Science and ICT, and Bio & Medical Technology Development Program (grant number NRF-2021M3E5E3080558) of the National Research Foundation (NRF) funded by the Ministry of Science & ICT and partially supported by the Brain Korea 21 Plus Program.

## Conflict of Interest

The authors declare that the research was conducted in the absence of any commercial or financial relationships that could be construed as a potential conflict of interest.

## Publisher’s Note

All claims expressed in this article are solely those of the authors and do not necessarily represent those of their affiliated organizations, or those of the publisher, the editors and the reviewers. Any product that may be evaluated in this article, or claim that may be made by its manufacturer, is not guaranteed or endorsed by the publisher.

## Footnotes

^1^ https://www.iedb.org/
