# Supplementary material for: Peptides Derived From S and N Proteins of Severe Acute Respiratory Syndrome Coronavirus 2 Induce T Cell Responses: A Proof of Concept for T Cell Vaccines
Source: Front Microbiol. 2021 Sep 24;12:732450. doi: 10.3389/fmicb.2021.732450 (PMC8498111; doi:10.3389/fmicb.2021.732450)
Supplement: Supplementary file 1 [file Data_Sheet_1.docx]

**Supplementary Materials**

**
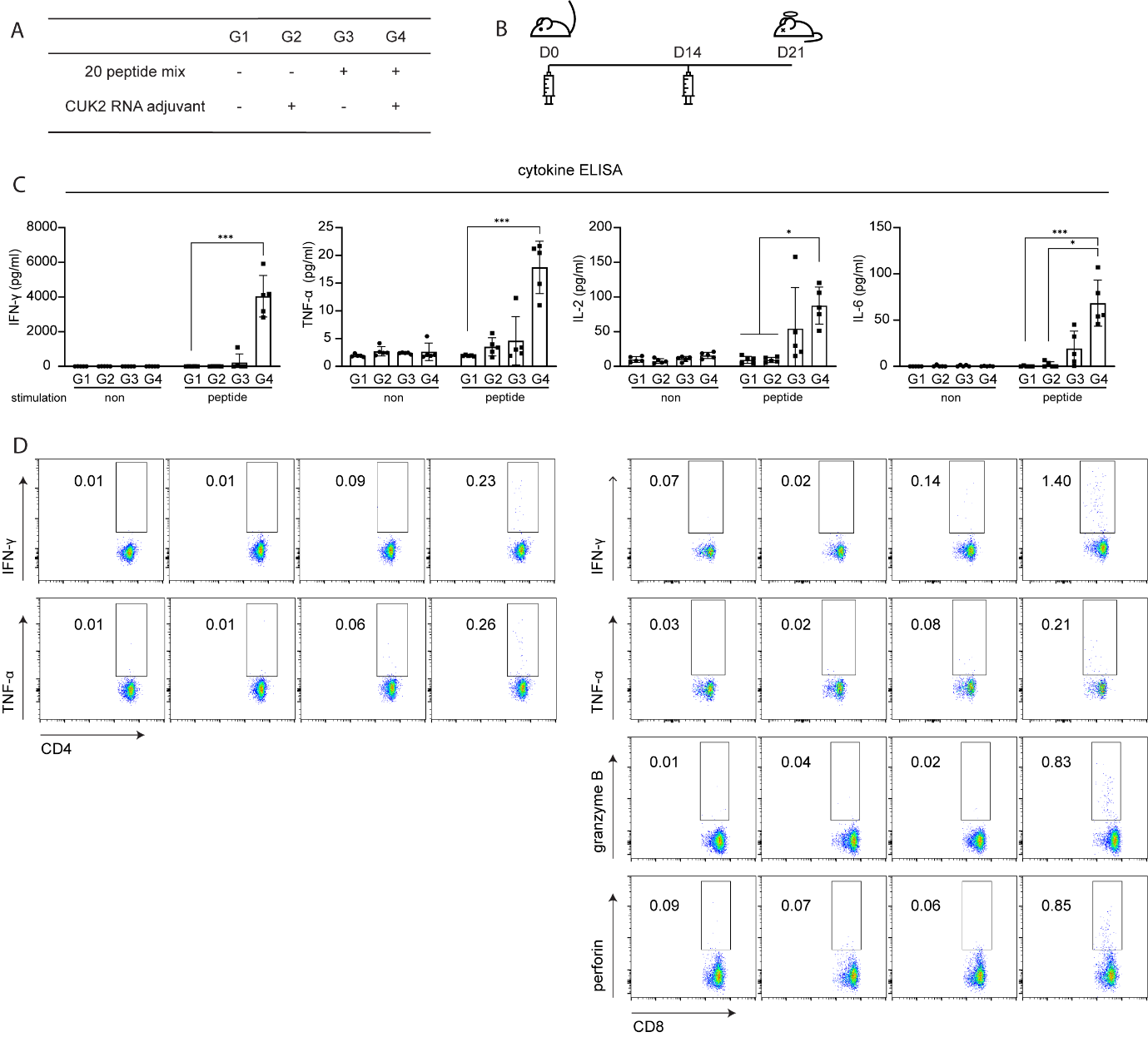
**

**Figure S1.** **Cytokine levels of splenocyte culture supernatants stimulated with the SARS-CoV-2 twenty-peptides mixture.** (A) Overview of the experimental groups. (B) Schedule of mice immunization; C57BL/6 mice were intramuscularly immunized at 2-week intervals, with two doses of the twenty SARS-CoV-2 peptides + CUK2 RNA adjuvant and sacrificed 1 week after the completion of the second immunization. Splenocytes from immunized mice were stimulated with twenty peptides mixture as described in material and methods. **(**C**)** The levels of IFN-γ, TNF-α, IL-2, and IL-6 in the culture supernatants of splenocytes were determined using ELISA. (D) Representative flow cytometry data showing CD4^+^IFN-γ, CD4^+^TNF-α, CD8^+^IFN-γ^+^, CD8^+^TNF-α, CD8^+^granzymeB^+^, CD8^+^Perforin^+^ cells. Data represent mean ± SD. *p < 0.05; ***p < 0.005.

**
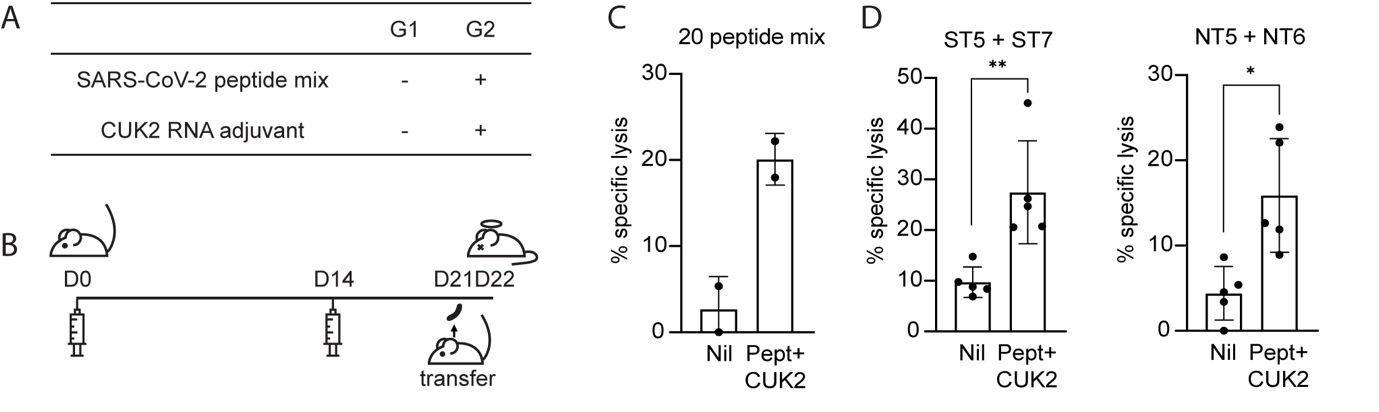
**

**Figure S2.** **Detection of SARS-CoV-2 peptide-specific CTL responses using in vivo CTL assays.** (A) Overview of the experimental groups. (B) Schedule of mice immunization. (C, D) Quantification of peptide-specific target cell lysis from flow cytometric analysis. Data represent mean ± SD. *p ≤ 0.05; **p ≤ 0.01.


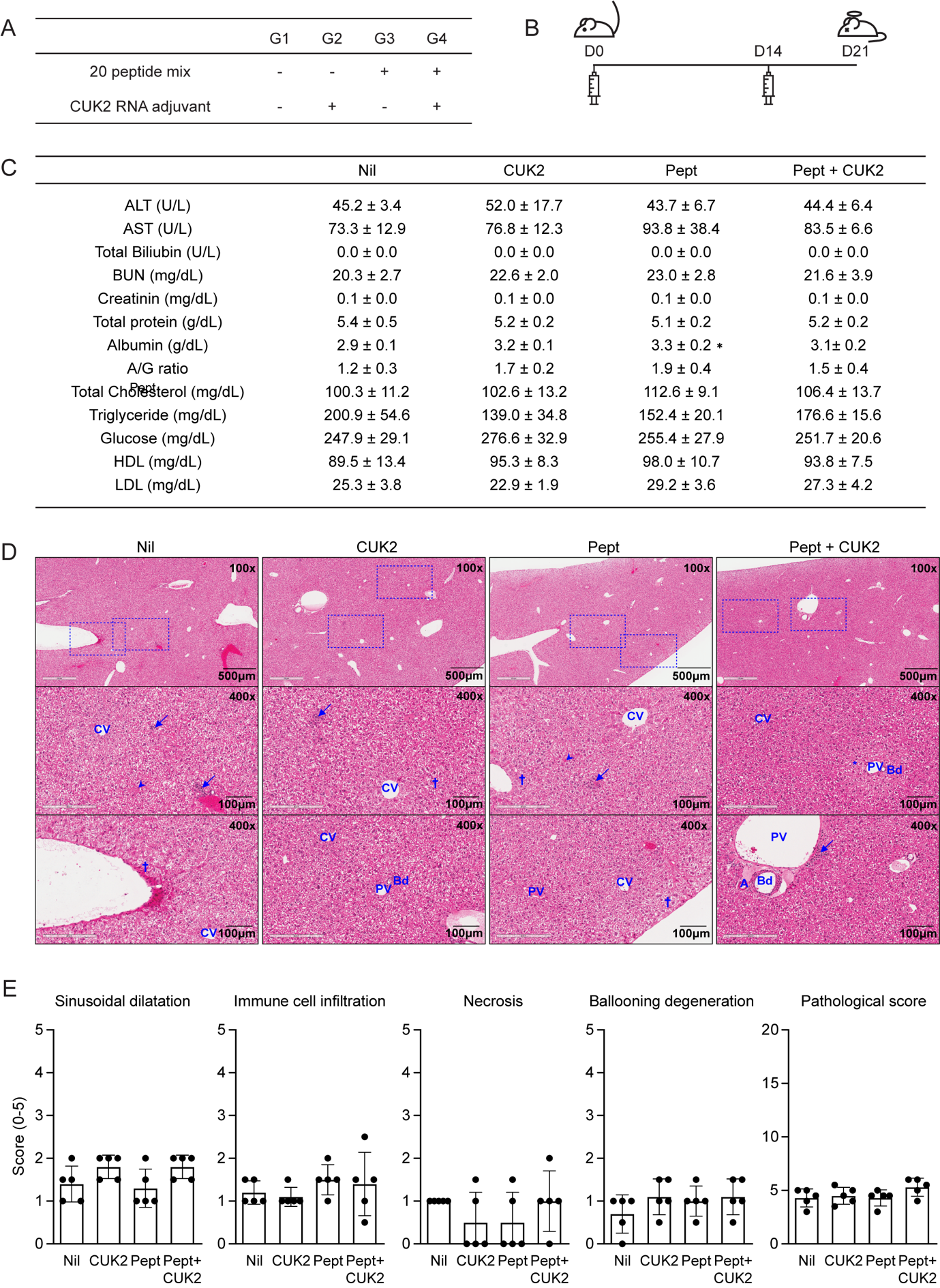


**Figure S3.** **SARS-CoV-2 peptide vaccine does not induce toxicity in C57BL/6 mice.** (A) Overview of the experimental groups. (B) Schedule of mice immunization. (**C**) Comparison of serum biochemical data. (D) Histological analysis of immunised mouse liver tissue samples. Tissue sections were stained using haematoxylin and eosin 1 week after the completion of the last immunization. (E) Histopathological scores of liver tissue samples were determined in a double-blinded manner (0, no lesions; 1, 1%–20%; 2, 21%–40%; 3, 41%–60%; 4, 61%–80%; 5, 81%–100%). CV, central vein; PV, portal vein; Bd, bile duct; A, hepatic artery. Arrows indicate immune cell infiltration; arrowheads indicate sinusoidal dilatation; the cross indicates ballooning degeneration; and the asterisk indicates necrosis. Data represent mean ± SD.
